# Low survival rate and muscle fiber-dependent aging effects in the McArdle disease mouse model

**DOI:** 10.1038/s41598-019-41414-8

**Published:** 2019-03-26

**Authors:** Alberto Real-Martinez, Astrid Brull, Jordi Huerta, Guillermo Tarrasó, Alejandro Lucia, Miguel Angel Martin, Joaquin Arenas, Antoni L. Andreu, Gisela Nogales-Gadea, John Vissing, Thomas O. Krag, Noemi de Luna, Tomàs Pinós

**Affiliations:** 1 Mitochondrial and Neuromuscular Disorders Unit, Vall d’Hebron Institut de Recerca, Universitat Autònoma de Barcelona, Barcelona, Spain; 2 Sorbonne Université, INSERM UMRS_974, Center of Research in Myology, 75013 Paris, France; 3 Faculty of Sport Sciences, Universidad Europea de Madrid, Madrid, Spain; 4 Mitochondrial and Neuromuscular Diseases Laboratory, 12 de Octubre Hospital Research Institute (i+ 12), Madrid, Spain; 5 Centro de Investigación Biomédica en Red de Enfermedades Raras (CIBERER), Madrid, Spain; 6 Grup de Recerca en Malalties Neuromusculars i Neuropediàtriques, Department of Neurosciences, Institut d’Investigacio en Ciencies de la Salut Germans Trias i Pujol i Campus Can Ruti, Universitat Autònoma de Barcelona, Badalona, Spain; 7 Copenhagen Neuromuscular Center, Department of Neurology, Rigshospitalet, University of Copenhagen, Copenhagen, Denmark; 8 Laboratori de Malalties Neuromusculars, Institut de Recerca Hospital de la Santa Creu i Sant Pau, Universitat Autònoma de Barcelona, Barcelona, Spain

## Abstract

McArdle disease is an autosomal recessive disorder caused by the absence of the muscle glycogen phosphorylase, which leads to impairment of glycogen breakdown. The McArdle mouse, a model heavily affected by glycogen accumulation and exercise intolerance, was used to characterize disease progression at three different ages. The molecular and histopathological consequences of the disease were analyzed in five different hind-limb muscles (*soleus*, *extensor digitorum longus*, *tibialis anterior*, *gastrocnemius* and *quadriceps*) of young (8-week-old), adult (35-week-old) and old (70-week-old) mice. We found that McArdle mice have a high perinatal and post-weaning mortality. We also observed a progressive muscle degeneration, fibrosis and inflammation process that was not associated with an increase in muscle glycogen content during aging. Additionally, this progressive degeneration varied among muscle and fiber types. Finally, the lack of glycogen content increase was associated with the inactivation of glycogen synthase and not with compensatory expression of the *Pygl* and/or *Pygb* genes in mature muscle.

## Introduction

McArdle disease (glycogen storage disease V; myophosphorylase deficiency; OMIM database number ♯232600; ORPHA: 368) is an autosomal recessive disorder caused by pathogenic mutations in the gene (*PYGM*) encoding the muscle isoform of glycogen phosphorylase (GP-M, also known as myophosphorylase)^1,2^. GP-M initiates the breakdown of muscle glycogen, leading to the release of glucose-1-phosphate in muscle fibers. Patients are unable to obtain energy from their muscle glycogen stores^3^, and present with exercise intolerance, usually in the form of reversible, acute crises of early exertional fatigue and contractures that can also be accompanied by rhabdomyolysis, as reflected by marked increases in serum levels of creatine kinase (CK) or even myoglobinuria (‘dark urine’)^4,5^. Naturally occurring or laboratory-generated animal disease models allow performing mechanistic studies that provide insights into the pathophysiology of a disorder. In this context, a knock-in McArdle mouse model with the most common *PYGM* pathogenic mutation (p.R50X) mutation was developed by our group^6^. The McArdle mouse mimics the phenotype found in patients, characterized by hyperCKemia, myoglobinuria, and poor exercise performance^6^. Glycogen levels in muscle of the mouse McArdle model are elevated several fold more than found in people with McArdle disease^6^. Several studies have been performed in the McArdle mouse model, analyzing the disease phenotype in young (8-week-old)^6–9^ or adult mice (20-week-old)^10–12^. In these studies, it was observed that muscle contractions were affected by structural degeneration due to glycogen accumulation and that glycolytic muscles fatigued prematurely^10^. Additionally, histological differences among glycolytic muscles were shown, as *tibialis anterior* (TA) muscles were invariably more damaged than *quadriceps* muscles, likely as a result of higher levels of glycogen metabolism in TA compared to *quadriceps*^10^. With respect to metabolic adaptive responses, *quadriceps* from McArdle mice presented significant changes in protein levels involved in glucose metabolism, while distal muscles such as TA, *extensor digitorum longus* (EDL) and *soleus* showed barely any change^11^. Additionally, differential structural damage was also observed among fiber types; while type I/IIa, IIa, IIa/IIx and IIx fibers from hind limb muscles presented evident signs of degeneration, type I fibers remained largely unaffected^10^. As disease phenotype progression and its molecular consequences during the aging process remain largely unknown, in the present study we wanted to perform a longitudinal characterization of the McArdle mouse model to determine if the massive glycogen accumulation in myofibers (*i*) increased with aging and *(ii)* caused progressive and differential increase in muscle and fiber type degeneration. Therefore, we analyzed and characterized the molecular and histopathological consequences of aging in five different hind-limb muscles [*soleus* (oxidative), and EDL, TA, *gastrocnemius* and *quadriceps* (glycolytic muscles)] from three cohorts of mice aged 8, 35 and 70 weeks. Furthermore, as we have previously observed an unusually high mortality of McArdle mice^10^, we also wanted to analyze the survival rate of these mice throughout their life-span.

## Material and Methods

### Ethical approval

All experimental procedures were approved by the *Vall d’Hebron* Institutional Review Board (protocol number 13/04 CEEA; 35/04/08) and were conducted in accordance with the European Convention for the Protection of Vertebrate Animals used for Experimental and Other Scientific Purposes (ETS 1 2 3) and Spanish laws (32/2007 and R.D. 1201/2005).

### Mice

Previously developed *p.R50X/p.R50X* knock-in McArdle mice, back-crossed for 10 generations to C57/6 J background, were used in this study^6,7^. To test for disease progression, three cohorts of mice aged 8, 35 and 70 weeks were used. All cohorts had N = 12 both for wild-type (WT) and McArdle (McA) mice, with the exception of 70-week-old McA mice where the number of studied mice were 6. All mice were killed by cervical dislocation immediately before dissection of the hindlimb muscles *soleus*, *gastrocnemius*, EDL, TA and *quadriceps*.

### Genotyping

Genotyping was performed for all post-weaning mice of the colony born between 01/01/2013 and 05/08/2018 (n = 2,088) and 38 embryos (E 14.5) according to a protocol previously described^6^.

### Survival curve

The survival curve was calculated for all the genotyped post-weaning mice of the colony that were born between 01/01/2013 and 05/08/2018 (n = 2,088). The Kaplan-Meier survival function, also known as the product-Limit estimator was used^13^. For each mouse, the following data were entered (GraphPad Prism version 6.00 for Windows, GraphPad Software, La Jolla, CA, www.graphpad.com): 1) X-values: time (number of days alive until an event or censoring occurred); 2) Y-values: Y value is “1” when the mouse died at the specified time, and “0” when the mouse’s data was censored at that time.

### Histology and immunohistochemistry

Dissected muscles were flash frozen in isopentane cooled in liquid nitrogen and stored at −80 °C until analysis. Twelve μm cryosections were stained with hematoxylin and eosin (H&E) for general histopathological evaluation as previously described^6^. Glycogen content was analyzed with periodic acid–Schiff (PAS) staining by sequentially incubating the sections with: periodic acid (Fisher Scientific; Hampton, NH) (0.5%) for 5 min, water wash, Schiff’s solution (Merck-Millipore, Burlington, MA) for 15 min, water for 1 min, alcohol-xylol dehydration and DPX mounting (Sigma-Aldrich, St. Louis, MO)^6^. For immunohistochemistry, sections were fixed in 10% buffered formalin (Sigma-Aldrich) and subsequently blocked in buffer (3% fetal calf serum in PBS) prior to staining. For damage, regeneration, inflammatory, fibrosis, and fiber type overview and analysis, sections were stained with their corresponding antibodies (see Supplementary Table). Finally, sections were stained with 4′,6-diamidino-2-phenylin- dole (DAPI) nucleic acid stain reactive (Invitrogen; Carlsbad, CA), for 5 min and mounted with ProLong^TM^ Gold Antifade reagent (Molecular probes; Eugene, OR). Images were taken using a FSX100 fluorescence microscope and the software FSX-BSW (Olympus; Tokyo, Japan).

### Central nuclei quantification

For the assessment of centrally nucleated fibers (CNF), sections were stained for laminin (for antibodies, see Supplementary Table) to distinguish individual muscle fibers and DAPI to visualize the nuclei. Total and CNF were counted manually, and the results were recorded using the cell counter plug-in from the image J software 1.37 version (NIH; Roth Bethesda, MO). Between 491 and 5,079 fibers (median 1682 fibers) from at least three different mice were counted per age and genotype. Results were expressed as the mean percentage of CNF per muscle. For enhanced visualization of nuclei staining surrounding the sarcoplasmic membrane [(peripheral nuclei (PN)], laminin-DAPI staining images were opened in TIFF format with the Adobe Photoshop program (Adobe^®^ Photoshop^®^ CS5 extended v.12.0, San Jose, CA, USA) and tone- and saturation-adjusted.

### Fiber size determination

Fiber size was calculated from laminin-DAPI stains using the minimal Feret’s diameter (Image J software ver. 1.37) to attain the fiber size least influenced by sample sectioning angle^14^. Between 240 and 1,444 fibers (median 459 fibers) from at least three different mice were counted per age and genotype.

### Fiber type staining and quantification

To identify individual and mixed fiber types, immunostains were made as triple stains for myosin heavy chain (MHC)I/MHCIIA/DAPI, MHCIIA/MHCIIX/DAPI, and MHCIIX/MHCIIB/DAPI (for antibodies, see Supplementary Table). The different fiber types were counted manually. Between 50 and 980 fibers (median 505 fibers) from at least three different mice were counted per age, genotype and fiber type. The results were recorded using the cell counter plug-in as described above and expressed as the percentage of (*i*) fiber types per muscle, and (*ii*) CNF per fiber type. For metabolic analyses, type I, IIa and I/IIa were grouped as oxidative fibers, while type IIx, IIa/IIx and IIx/IIb as glycolytic fibers^15^. The results were expressed as the percentage of each group relative to total number of fibers counted.

### mRNA analysis

Total RNA was obtained from *gastrocnemius* muscle as previously described^16^ following the manufacturer instructions of TRIzol (Invitrogen). RNA was treated with DNase I, amplification grade (Invitrogen) to eliminate any traces of DNA. Complementary DNA was synthesized from RNA using the high-capacity complementary DNA archive kit (Applied Biosystems, Foster City, CA), which uses random primers. We used real-time PCR, with TaqMan fluorogenic probes in a 7900 Real-Time PCR System (Applied Biosystems) to assess *gastrocnemius* RNA levels of: (i) *Pygm* gene (Mm00478582_m1); (ii) glycogen phosphorylase, brain isoform (*Pygb*) gene (Mm00464080_m1); and (iii) glycogen phosphorylase, liver isoform (*Pygl*) gene (Mm00500078_m1). Results were normalized to peptidylprolyl isomerase A (cyclophilin A, Ppia) gene messenger RNA levels (probe Mm02342430_g1).

### Western blot analysis

Muscle samples from *gastrocnemius* and TA were homogenized using Pellet pestles Cordless motor (Sigma-Aldrich) in cold homogenization buffer (Tris-Hcl 20 mM, NaCl 150 mM and Triton X100 1%) and centrifuged at 10,000 *g* for 10 min at 4 °C. Proteins (30 μg) were resolved on Criterion^TM^ TGX^TM^ 4–15% Precast Midi gels (Biorad; Hercules, CA) at 150 V for 90 min and blotted to a polyvinylidene difluoride membrane (Immun-Blot^®^ PVDF membrane, Biorad) using the Trans-Blot® SD Semi-dry Transfer Cell (Biorad) at 20 V for 50 min. Membranes were incubated in primary antibodies overnight at 4 °C and in secondary antibodies for 3 hours at room temperature (Supplementary Table). Ponceau S staining (Sigma-Aldrich) was used as a loading control for all the membranes. Membranes were developed with Immobilon Western Chemiluminiscent HRP Substrate (Merck-Millipore) and images obtained with a Fujifilm LAS 3000 imager (R&D Systems; Minneapolis, MN) and quantified with Image J, version 1.37.

### Muscle glycogen

One hundred and fifty mg of tissue were boiled for 30 min with 30% KOH and, subsequently, 1.2 volumes of 95% ethanol were added to precipitate glycogen. After a centrifugation step (25 min at 840 *g*), the glycogen pellet was resuspended in 0.3 ml of water. Next, 0.1 ml of 5% phenol was added to 0.1 ml of sample and treated with 0.5 ml of H_2_SO_4_ (to hydrolyze glycogen to glucose). The mixture was allowed to stand for 30 min at room temperature and the glucose released was measured spectrophotometrically at 490 nm. A standard curve made with glycogen purified from rabbit liver (Sigma-Aldrich), ranging from 0.1 to 0.8 mg mL^−1^, was processed in parallel. The results were expressed as mg glycogen (g wet tissue)^−1,^^17^.

### Statistics

All statistical analyses were performed using the GraphPad Prism software. To analyze the aging effects on muscle fiber size determination (mFd), as well as the number of mice per litter in the colony breeding (normally distributed values), One-Way ANOVA with post-hoc Tukey Honestly Significant Difference (HSD) test was applied. To analyze the aging effects when values were not normally distributed (% CNF, % MHCe+, % fiber types, PN per fiber, glycogen content, mRNA and protein relative levels), the non-parametric Kruskal-Wallis One*-*Way ANOVA with multiple comparisons test (*Dunn’s test*) was used. To analyze the genotype effects in normally distributed values, a Student t-test was applied, whereas the non-parametric Mann-Whitney U-test was used when values were not normally distributed. To evaluate the distinct survival rates of each genotype (Kaplan- Meier curve), the log-rank (Mantel-Cox) test was used. To compare obtained versus expected mouse genotypes we used the Fisher’s exact test (two-group comparisons, *i.e*., WT x HTZ and HTZ x McA breedings) or the Chi-square test (three-group comparisons, *i.e*., HTZ x HTZ breedings).

## Results

### Perinatal mortality

During the period from January 2013 to May 2018 a total of 139 matings of the McArdle mouse model colony were performed. From these, 19 were wild-type (WT) x heterozygous (HTZ), 47 HTZ x HTZ, 64 McArdle (McA) x HTZ and 9 McA x McA matings, resulting in a total of 583 litters and 2,227 mice [1,133 males (51%) and 1,094 females (49%)] (Table 1). The number of offspring was significantly different among the distinct matings, showing that a higher presence of the mutant p.R50X allele in parents significantly reduced the number of mice per litter in the offspring (Table 1); additionally, the presence of the p.R50X allele in parents was also associated with an increased percentage of litters with a 100% mortality (Table 1). Furthermore, when the offspring mice originating from HTZ x HTZ and McA x HTZ matings were genotyped we observed a clear decrease in the proportion of McA offspring compared to the expected 25% and 50%, respectively, from Mendelian inheritance (Table 2). Genotyping of 38 embryos originating from HTZ x HTZ mating demonstrated a normal Mendelian distribution of McA mouse embryos (Table 2). These results suggest that McA mice have high perinatal mortality.

**Table 1:** McArdle mice colony breeding results between 2013 and 2018. Abbreviations. (M): Males; (F): Females; N.A: Not applicable.

	Breedings (2013–2018)							
	WT x HTZ		HTZ x HTZ	McA x HTZ		McA x McA	Statistics	Total
	WT (M) x HTZ (F)	HTZ (M) x WT (F)	HTZ (M) x HTZ (F)	McA (M) x HTZ (F)	HTZ (M) x McA (F)	McA (M) x McA (F)		
Total number of breedings	9	10	47	58	6	9	N.A	**139**
Total number of litters	17	41	273	179	43	30	N.A	**583**
Total number of mice	103	189	1149	617	130	39	N.A	**2227**
Total number of males	45	102	578	325	64	19	N.A	**1133**
% of males	43,7	54,0	50,3	52,7	49,2	48,7	N.A	**50,9**
Total number of females	58	87	571	292	66	20	N.A	**1094**
% of females	56,3	46,0	49,7	47,3	50,8	51,3	N.A	**49,1**
Mice/litter	**6,06** (±3,75)	**4,61** (±3,69)	**4,21** (±3,33)	**3,45** (±2,75)	**3,02** (±2,15)	**1,30** (±1,74)	p < 0.0001	**3,77** (±1,61)
	**5,03** (±3,73)		**4,21** (±3,33)	**3,36** (±2,64)		**1,30** (±1,74)		
Nº litters with 100% mortality	3	13	61	48	7	14	N.A	**146**
% litters with 100% mortality	**5,5**		**22,3**	**24,8**		**35,9**	N.A	**22,1**

To calculate the statistical significance of the number of mice per litter the One-Way ANOVA with post-hoc Tukey Honestly Significant Difference (HSD) test was applied.

**Table 2:** McArdle mice colony genotyping results between 2013 and 2018. Abbreviations.

Breedings	Post-weaning genotyping									Embryo genotyping		
	WT x HTZ			HTZ x HTZ			HTZ x McA			HTZ x HTZ		
Offsprings	WT	HTZ	McA	WT	HTZ	McA	WT	HTZ	McA	WT	HTZ	McA
Nº mice per genotype	96	137	N.A	368	718	67	N.A	580	122	12	17	9
Genotype %	**41.2**	**58.8**	N.A	**31.9**	**62.3**	**5.8**	N.A	**82.6**	**17.4**	**31.5**	**44.7**	**23.6**
Expected Genotype %	50	50	N.A	25	50	25	N.A	50	50	25	50	25
**Statistics**	**Fisher’s test p = 0.035**			**Chi-square test p** < **0.001**			**Fisher’s test p** < **0.001**			Chi-square test p = 0.864		

N.A: Not applicable. To compare obtained *versus* expected mouse genotypes Fisher’s exact test (two-group comparisons, *i.e*., WT x HTZ and HTZ x McA breedings) and Chi-square test (three-group comparisons, *i.e*., HTZ x HTZ breedings) were used.

### Post-weaning mortality

**Figure 1:**
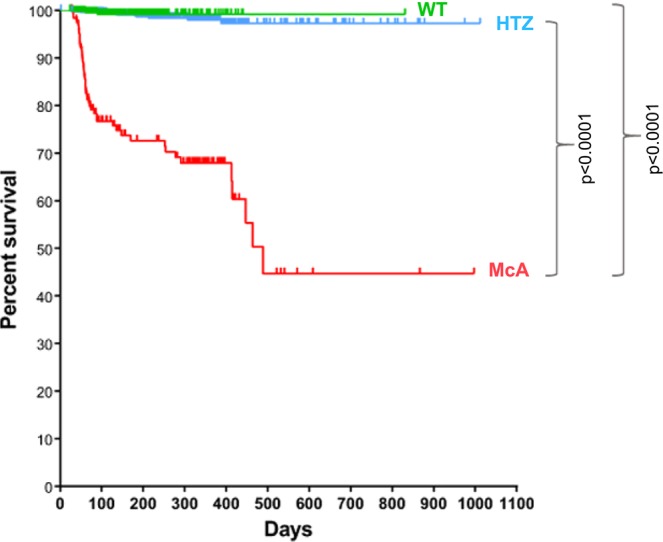
Kaplan and Meier survival plot for the wild-type (WT), heterozygous (HTZ) and McArdle (McA) mice born between 01/01/2013 and 05/08/2018. There was a significant reduction in the survival rate of McA with respect HTZ and WT [p < 0.0001; log-rank (Mantel-Cox test)]. From 464 WT, 461 were censored and 3 spontaneous deaths (events) occurred in 831 days; from 1435 HTZ, 1420 were censored and 15 events occurred in 1012 days; finally, from 189 McA, 137 were censored and 52 events occurred in 997 days.

A survival curve for all post-weaning genotyped mice (n = 2,088; WT = 464; HTZ = 1,435; McA = 189; including males and females) was calculated. There was a significant reduction in the survival rate of McA mice compared with WT and HTZ (Fig. 1); the accumulative survival for WT was 99.3% in a serial time of 831 days, 98.9% for HTZ in a serial time of 1012 days, and only 44.7% in McA mice in a serial time of 997 days (Fig. 1).

### Degeneration, fibrosis and inflammation

**Figure 2:**
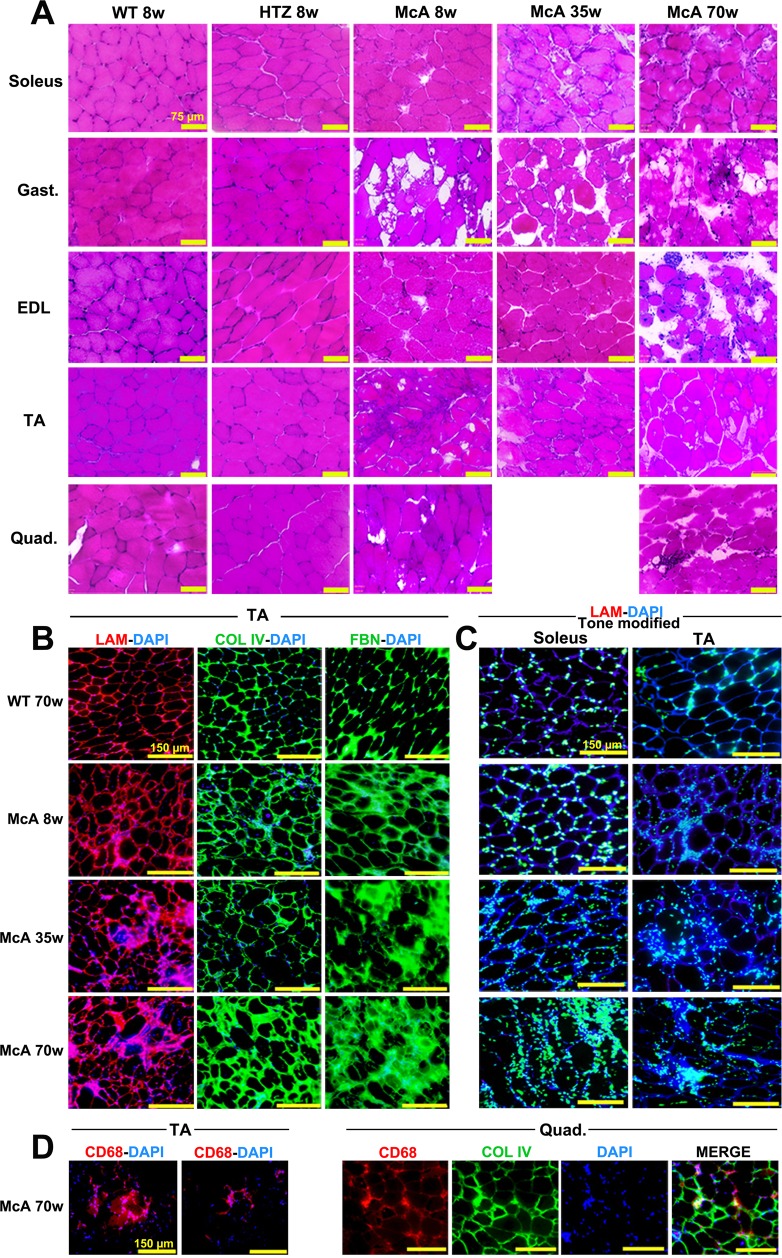
Histological characterization of McArdle mice. (**A**) H&E staining of the *soleus*, *gastrocnemius*, *extensor digitorum longus* (EDL), *tibialis anterior* (TA) and *quadriceps* muscles from 8-week-old wild-type (WT) and heterozygous (HTZ) and 8, 35 and 70-week-old McArdle (McA) mice. All scale bars correspond to 75 µm. (**B**) Laminin-DAPI, Col IV-DAPI and FBN-DAPI stains of TA muscles from 8, 35 and 70-week-old McA and 70-week-old WT. All scale bars correspond to 150 µm. (**C**) Tone modified (Adobe^®^ Photoshop^®^ tone and saturation adjustments were set at −105 and +25, respectively) laminin-DAPI stain of *soleus* and TA muscles from 70-week-old WT and 8, 35 and 70-week-old McA. Due to the tone modification laminin staining has become dark blue, whereas DAPI staining has become light blue/green and nuclei are much more highlighted. All scale bars correspond to 150 µm. (**D**) CD68 (pan-macrophage marker)-DAPI staining of TA from 70-week-old McA and CD68, Col IV and DAPI stains of *quadriceps* from 70-week-old McA mice.

Histopathological evaluation of *soleus*, EDL, *gastrocnemius*, TA and *quadriceps* from 8, 35 and 70-week-old McA mice using H&E-staining revealed several muscle fibers in disarray, variations in fiber size, with large intra-fiber voids and some CNF and an increase in the extracellular matrix area, that became more apparent in 70-week-old mice **(**Fig. 2A). Additionally, detailed images from 8, 35 and 70-week-old McA EDL muscle showed the presence of muscle regions with a high proportion of CNF along with an increase in the number of central nuclei per fiber in the EDL of 70-week-old mice (Supplementary Fig. 1). To assess whether this age-related increase in the extracellular matrix area was associated with fibrosis, muscle sections were stained with laminin, collagen IV (Col IV) and fibronectin (FBN) antibodies. TA from 8, 35 and 70-week-old McA mice clearly demonstrated fibrosis compared to age-matched WT mice (Fig. 2B and Supplementary Fig. 2A). Furthermore, in order to determine whether this finding was also present in other muscle types, laminin-fibronectin double stain as well as collagen I (Col I) and Col IV single stains were performed in EDL, *quadriceps* and *soleus* from WT and McA mice; in these muscles, the laminin and fibronectin stains were specially marked in the EDL and *soleus* of 70-week-old McA, while in *quadriceps* the increase was less evident (Supplementary Fig. 2B). Additionally, using laminin-DAPI stain, we observed an increase in the number of nuclei staining surrounding the sarcoplasmic membrane of TA and *soleus* muscle fibers, which was already apparent in 8-week-old McA mice, with a subtle increase in 35 and 70-week-old McA mice (Fig. 2C and Supplementary Fig. 3). To determine whether this increase was associated with an increment in the presence of inflammatory cells, we stained TA and *quadriceps* muscles with the CD68 macrophage marker^18^, and observed that high intensities of CD68 staining co-localized with the presence of multiple nuclei outside the sarcoplasmic membrane (Fig. 2D), reflecting the presence of an inflammatory response. Additionally, CD68, Col IV and DAPI stains co-localized in the quadriceps of 70-week-old McA mice, suggesting that fibrosis and inflammation are associated processes in the skeletal muscle of these mice (Fig. 2D).

### Age-dependent increase in fiber degeneration

**Figure 3:**
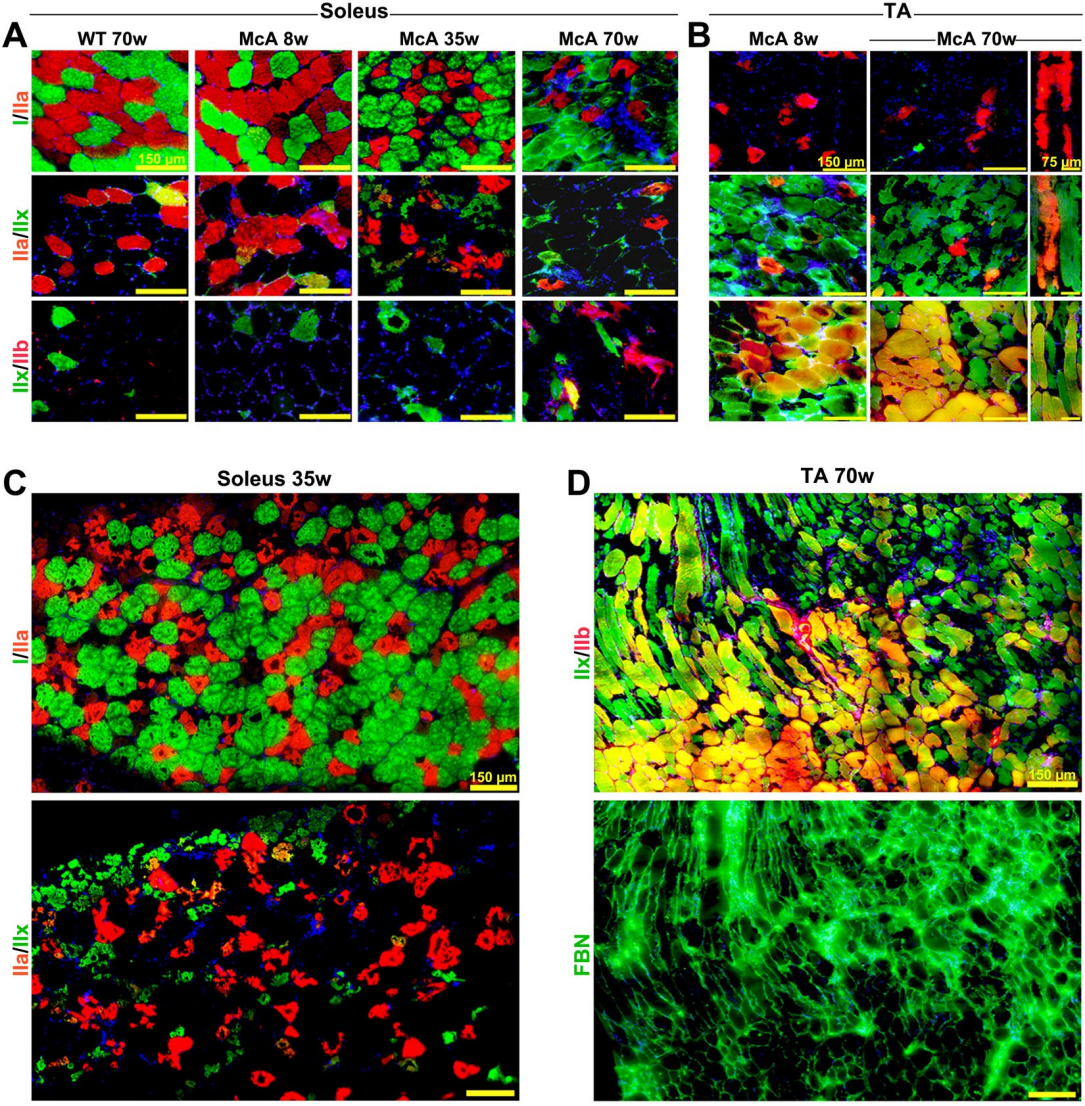
Age-dependent increase of IIa and IIx fibers atrophy. (**A**) Fiber type stains for myosin heavy chain (MHC) I, IIa, IIx, and IIb as overlapping double stains in *soleus* muscle from McArdle (McA) mice demonstrate that type I fibers are not affected structurally by glycogen accumulation in any of the ages analyzed, whereas type IIa, IIa/IIx mixed, and IIx fibers present an age-dependent increase in atrophy. All scale bars correspond to 150 µm. (**B**) MHC double stain in *tibialis anterior* (TA) muscle shows atrophy in IIa, IIx and mixed IIa/IIx fibers in 8-week-old McArdle (McA) mice, which is further exacerbated in 70-week-old McA mice. Mixed IIx/IIb fibers seems to be protected from atrophy. Longitudinal sections are also shown. (**C**) Consecutive *soleus* sections from 35-week-old McA mice stained for I/IIa and II/IIx fibers showing almost intact type I fibers but highly degenerated IIa, IIx and IIa/IIx fibers. Scale bars correspond to 150 µm. All scale bars correspond to 150 µm, while in longitudinal sections the scale bars correspond to 75 µm. (**D**) Two consecutive sections of TA muscle from 70 wo McA mice stained for IIx/IIb fibers and fibronectin shows the presence of fibrosis in muscle regions enriched in IIx fibers and its absence in IIx/IIb enriched sections. In (**A**–**D**) DAPI was used to stain cell nuclei.

Muscle fiber types IIa and IIx have previously been found to be the most degenerated muscle fibers in 20-week-old McA mice due to massive intermyofibrillar and subsarcolemmal glycogen accumulation disturbing the ultrastructure^10^. In the present study, IIa and IIx fibers degeneration was observed in *soleus* muscle of 8-week-old McA mice, which was further exacerbated in 35- and 70-week-old McA animals (Fig. 3A,C); interestingly, type I fibers presented almost normal morphology in the three age groups (Fig. 3A,C). Similarly, degeneration of IIa, IIx and IIa/IIx fibers was already observed in TA muscle from 8-week-old McA mice, although it was more pronounced in 70-week-old mice (Fig. 3B). Additionally, as previously reported^10^, mixed IIx/IIb fibers were less affected than IIx fibers alone (Fig. 3D). Of note, higher accumulations of the extracellular matrix protein fibronectin were observed in TA regions where IIx fibers were predominant, while in areas with IIx/IIb fibers, less fibronectin staining was seen (Fig. 3D); thus, an inverse correlation between fibrosis and presence of IIx/IIb fibers might exist.

### Muscle regenerates differentially depending on fiber type composition

**Figure 4:**
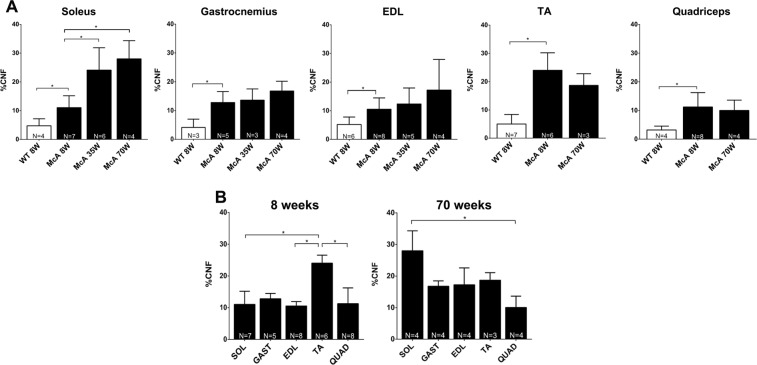
Quantification of centrally nucleated fibers (CNF) among the different muscles and ages. (**A**) Percentage of CNF from the total amount of fibers. White columns correspond to wild-type (WT) mice whereas black columns correspond to McArdle (McA) mice. Only WT values from 8 wo mice are shown, as values WT values are similar among the three different ages. Each column represents the percentage of CNF resulting from the mean value obtained from 3 to 8 mice. The total number of mice used (N) is indicated at the bottom of each column. In each column between 491 (minimum) and 5,079 (maximum) fibers were counted (median 1682 fibers). Error bars correspond to standard deviation (SD). (**B**) Percentage of CNF in the different muscles of 8 and 70-week-old McA mice. For each age, muscles are represented together. Error bars correspond to SD. The non-parametric Kruskal-Wallis One-Way ANOVA with multiple comparisons test (Dunn’s test) was used for statistical analyses. Symbols: *p < 0.05.

In order to assess whether McA mice muscles were progressively affected by the excess of glycogen, we quantified the presence of CNF as a marker for the ongoing degeneration/regeneration cycles known to exist in McA mouse muscles^10^. The percentage of CNF was superior in McA vs. WT in the *soleus*, *gastrocnemius*, EDL and TA in the three age groups (Fig. 4A); additionally, there was an age-dependent increase in the mean percentage of CNF in the *soleus* of the McA mice (8-, 35- and 70-week-old: 11, 24 and 28%, respectively) that was not observed in *gastrocnemius*, EDL, TA and *quadriceps* (Fig. 4A). Thus, while TA showed the highest proportion of CNF in 8-week-old McA mice (24%), the *soleus* muscle presented the highest percentage in 70-week-old McA animals (28%) (Fig. 4B).

### Increased number of CNF in type I and IIx/IIb fibers during aging

**Figure 5:**
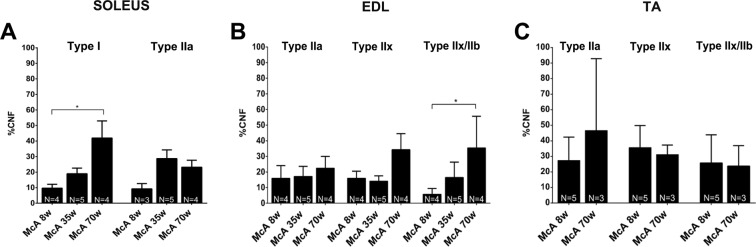
Quantification of central nucleated fibers (CNF) in *soleus*, EDL and TA fiber types. (**A**) Percentage of CNF in *soleus* type I and IIa fibers. In each column between 223 (minimum) and 980 (maximum) fibers were counted (median 612 fibers). (**B**) Percentage of CNF in EDL type IIa, IIx and IIx/IIb fibers. In each column between 177 (minimum) and 651 (maximum) fibers were counted (median 413 fibers). (**C**) Percentage of CNF in TA type IIa, IIx and IIx/IIb fibers. In each column between 50 (minimum) and 930 (maximum) fibers were counted (median 520 fibers). The N at the bottom of each column represents the number of mice used to calculate the mean values. Error bars correspond to SD. The non-parametric Kruskal-Wallis One-Way ANOVA with multiple comparisons test (Dunn’s test) was used for statistical analyses. Symbols: *p < 0.05.

To assess if specific fiber types were more prone to degeneration, the percentage of CNF was quantified within the different fiber types in one oxidative muscle (*soleus*) and two glycolytic muscles (EDL and TA). In *soleus* muscle, CNFs were increased in type I fibers between 8 and 70-week-old McA mice, whereas no increase was observed in IIa fibers (Fig. 5A). In the EDL muscle, there was a significant increase in the percentage of CNF in type IIx/IIb fibers between 70 and 8-week-old McA mice (Fig. 5B), In TA, no change in CNF was found between the different ages (Fig. 5C).

### Co-localization of MHCe with IIa, IIa/IIx and IIx fibers

**Figure 6:**
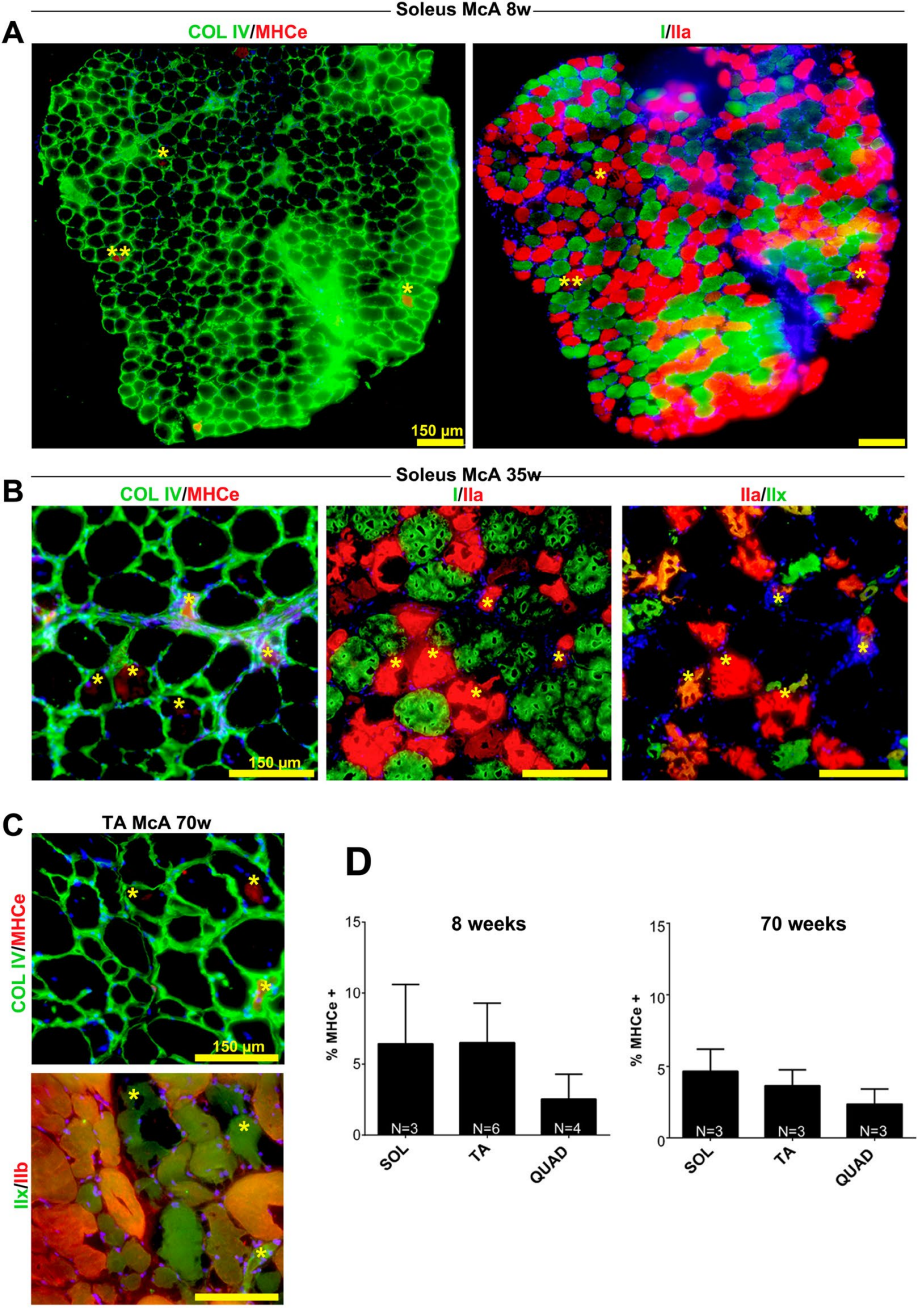
Co-localization of embryonic myosin heavy chain (MHCe) with IIa and IIx fibers. (**A**) Two consecutive *soleus* sections from 8-week-old McArdle (McA) mice were stained for Collagen IV and MHCe in the first section, and MHC I and IIa in the second section. All detected MHCe positive fibers co-localized with MHC IIa staining. (**B**) Three consecutive soleus sections from 35-week-old McA mice were double stained for collagen IV/MHCe, MHCI/IIa and MHCIIa/IIx showing a colocalization of the MHCe and IIa stainings. (**C**) Two consecutive TA sections were double stained for Collagen IV and MHCe in the first section and MHCIIx and IIb in the second section, showing co-localization of the MHCe and MHCIIx stains. In all cases yellow asterisks mark identical fibers among sections and scale bars correspond to 150 µm. (**D**) Percentage of MHCe positive fibers in *soleus*, *tibialis anterior* (TA) and *quadriceps* of 8 and 70-week-old McA mice. N is the number of mice used to calculate the mean values. In each column between 619 (minimum) and 2250 (maximum) fibers were counted (median 1462 fibers). Error bars correspond to standard deviation (SD). The non-parametric Kruskal-Wallis One-Way ANOVA with multiple comparisons test (Dunn’s test) was used for statistical analyses.

We next analyzed the expression of the embryonic myosin heavy chain (MHCe), a marker of ongoing (acute) regeneration in McA mice through aging and different muscles/fiber types. In this regard, McA mouse muscle sections were double stained for Col IV and MHCe and for type I/IIa/IIx fibers. Both in *soleus* from 8 and 35-week-old McA mice and TA from 70-week-old McA mice, MHCe was only found in types IIa, IIa/IIx and IIx, but not in type I fibers. (Fig. 6A–C). Additionally, we also observed that there was a general trend towards a decrease in the number of MHCe positive fibers during aging in all the three muscles analyzed **(**Fig. 6D).

### Fiber size and fiber type composition

**Figure 7:**
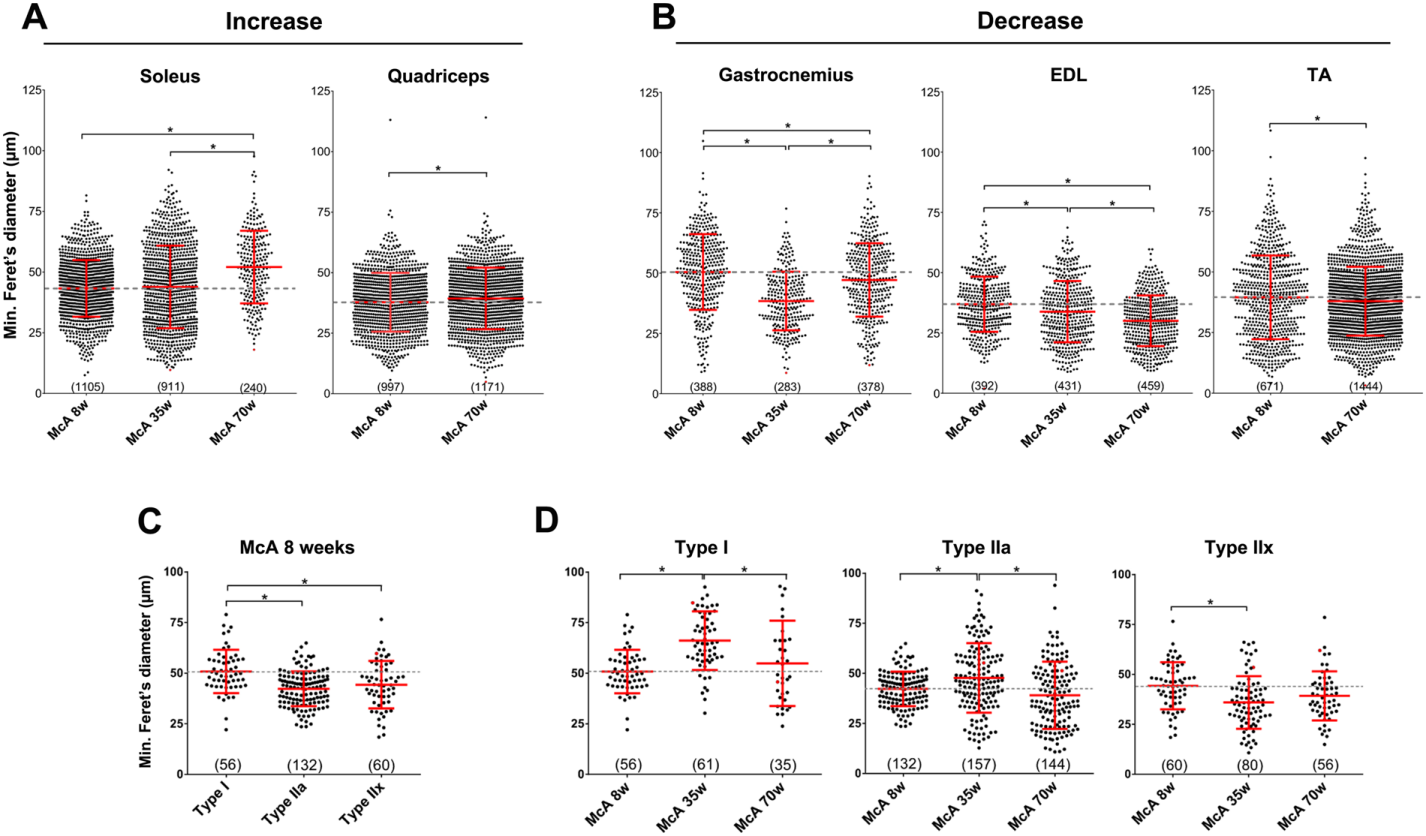
Fiber size determination using the minimum Feret diameter (mFd). (**A**) Fiber size increase in *soleus* and *quadriceps* between 8 and 70-week-old McA mice. (**B**) Fiber size decrease in *gastrocnemius*, EDL and TA between 8 and 70-week-old McA mice. (**C**) Type I, IIa and IIx *soleus* fiber size from 8-week-old McA mice. (**C**) Type I *soleus* fiber size increase and IIa and IIx fiber size decrease between 8 and 70-week-old McA mice. Data is represented as a Scatter Plot. Each dot represents the mFd of a single fiber. A grey dot-line indicates the mean value of McA 8-week-old mice in each muscle. For each plot the mean value and the SD are also indicated in red. Below each plot is indicated the total amount of fibers (between brackets) counted. Between 3 to 5 mice were used in each column. One-Way ANOVA with post-hoc Tukey Honestly Significant Difference (HSD) test was used for statistical analyses. Symbols: *p < 0.05.

To determine whether muscle degeneration affected muscle fiber size in McA mice, mFd was measured in the different muscles and age groups; while there were ~18% and ~4% mean fiber size increases in *soleus* and *quadriceps*, respectively (Fig. 7A), between 70 and 8-week-old McA mice, ~4, ~6 and ~19% mean fiber size decreases were observed in TA, *gastrocnemius* and EDL, respectively, between the same ages (Fig. 7B). These results suggest that among the analyzed muscles, *soleus* and *quadriceps* fibers are the least affected by McA disease during aging process in terms of fiber size. To further confirm the specific degeneration of IIa and IIx fibers during aging, we measured the mFd for each specific fiber type in the *soleus* of McA mice and observed that type I fibers were significantly larger than IIa and IIx fibers in 8-week-old McA animals (Fig. 7C). Additionally, while there was an ~8% increase in type I fiber size between 8 and 70-week-old McA mice, 7.5 and 11% decreases in type IIa and IIx fiber size, respectively, were observed between the same ages in the *soleus* of McA mice (Fig. 7D). These results suggest that repeated episodes of muscle damage and subsequent regeneration over mice life do not affect type I fiber size, but clearly reduce that of type IIa and IIx fibers. In *soleus*, EDL TA muscle we did not detect any changes in fiber type composition between the different ages and genotypes (Supplementary Fig. 4A–C). Next, we wanted to determine whether the overall muscle metabolism was affected. Fiber types were grouped either as oxidative (I, IIa and I/IIa) or glycolytic (IIx, IIa/IIx and IIx/IIb). While no significant changes were observed in both TA and EDL glycolytic muscles (Supplementary Fig. 5B,C), the *soleus* of McA mice demonstrated a progressive increase in the number of glycolytic fibers and a decrease in oxidative fibers with age, opposite of what was found in *soleus* of WT mice. (Supplementary Fig. 5A).

### No progressive accumulation of glycogen in McArdle mice

**Figure 8:**
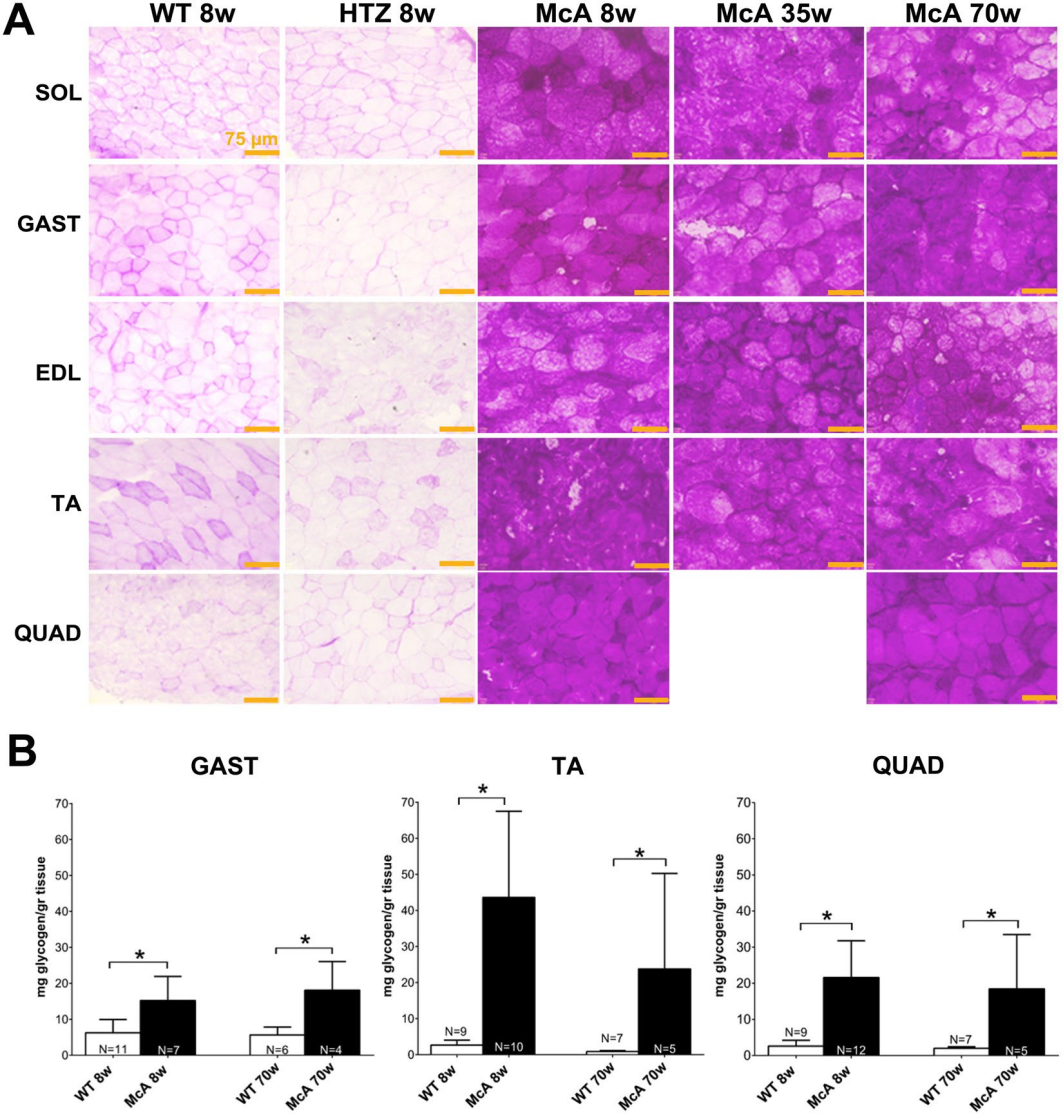
Histological and biochemical determination of glycogen in different muscles and ages of McArdle (McA) mice. (**A**) PAS staining of *soleus*, *gastrocnemius*, *extensor digitorum longus* (EDL), *tibialis anterior* (TA) and *quadriceps* in 8-week-old wild-type (WT) and heterozygous (HTZ) mice as well as in 8, 35 and 70-week-old McA mice. All the scale bars correspond to 150 µm. PAS staining was markedly increase in all sections from McA mice. (**B**) Biochemical determination of glycogen in TA, *quadriceps* and *gastrocnemius* of 8 and 70 wo WT and McA mice. N indicates the number of muscles used to calculate the mean values. Error bars correspond to SD. The non-parametric Kruskal-Wallis One*-*Way ANOVA with multiple comparisons test (*Dunn’s test*) was used for statistical analyses. Symbols: *p < 0.05.

**Figure 9:**
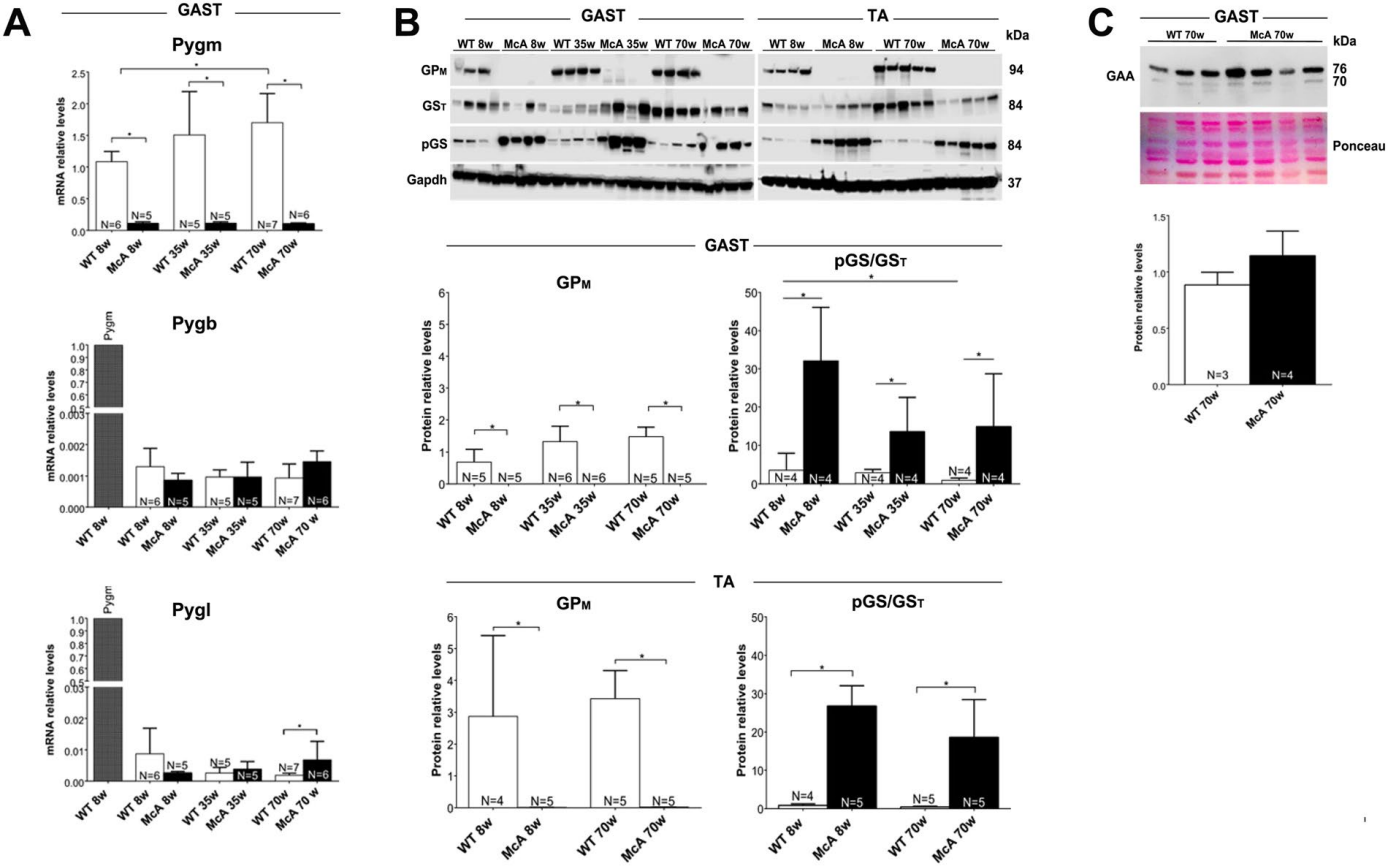
Expression studies of genes involved in glycogen synthesis and degradation. (**A**) Quantitative PCR analysis of the different glycogen phosphorylase isoforms in the *gastrocnemius* muscle. In the *Pygb* and *Pygl* studies, the relative *Pygm* expression values in one *gastrocnemius* muscle is shown. (**B**) Western blot analysis of GP_M_, total GS (GS_T_) and phospho serine 641 GS (pGS_Ser 641_) in *gastrocnemius* and *tibialis anterior* (TA) muscles. Glyceraldehyde-3-Phosphate Dehydrogenase (*Gapdh*) was used as a loading control. The blot images were obtained with Fujifilm LAS 3000 imager. These are cropped images. Uncropped original images can be found in Supplementary Fig. 5. The relative quantification for GP_M_ protein results from GP_M_/Gapdh ratio after image J quantification as well as the relative quantification of the phosphorylated fraction of (inactive) GS protein. This quantification results from pGS/GS_T_ ratio after image J quantification. (**C**) Alpha acid glucosidase protein levels in 70 wo WT and McA mice determined by western blot. Ponceau staining was used for loading normalization. Error bars correspond to standard deviation (SD). The non-parametric Kruskal-Wallis One*-*Way ANOVA with multiple comparisons test (*Dunn’s test*) was used for statistical analyses. Symbols: *p < 0.05.

We observed that glycogen levels were highly increased at all ages and muscles of McA mice compared with WT or HTZ using PAS stain (Fig. 8A). Biochemical quantification of glycogen in TA, *quadriceps* and *gastrocnemius* muscles in 8 and 70-week-old mice demonstrated that muscle glycogen levels were significantly higher in McA mice compared to WT (Fig. 8B). However, no difference in glycogen levels were found between 8 and 70-week-old McA mice in any of the three muscles examined **(**Fig. 8B). We analyzed the glycogen synthase (GS) expression and activation levels, as well as the expression levels of the of the different glycogen phosphorylase isoforms in *gastrocnemius* and TA muscles to determine if any changes were responsible for the absence of progressive glycogen accumulation. Using quantitative PCR, no significant change in *Pygm, Pygb and Pygl* mRNA levels were detected in *gastrocnemius* between 8 and 70-week-old McA mice (Fig. 9A). Additionally, there was a complete absence of GP-M protein in *gastrocnemius* and TA from McA mice in the three ages analyzed (Fig. 9B). A significant increase in the phosphorylation (and thus inactivation) of GS in McA animals compared with WT in both *gastrocnemius* and TA was observed (Fig. 9B). Thus, to further assess whether the absence of progressive glycogen accumulation could be associated with an increase in lysosomal glycogen degradation we compared acid-alpha glucosidase (GAA) protein levels between 70-week-old WT and McA mice, and no changes in GAA protein levels were found **(**Fig. 9C).

## Discussion

Since the publication in 2012 of the article describing the development and characterization of the McArdle mouse model^6^, several studies analyzing the biological consequences of GP-M deficiency in this model have been reported^7–12^. None of these studies accounted for the potential effects of aging on disease progression and its molecular consequences. Therefore, in the present study, we have performed a longitudinal characterization of the McArdle mouse model and have analyzed its survival rate. The most important observations that we have obtained are: (*i*) McA mice present high perinatal and post-weaning mortality; (*ii*) there is a lack of glycogen content increase in McA mice during aging (*iii*) there is a progressive muscle degeneration, fibrosis and inflammation; (*iv*) *soleus* (oxidative) and *quadriceps* (glycolytic) are histologically less affected by disease progression than *gastrocnemius*, EDL and TA (glycolytic muscles); (*v*) there is a progressive degeneration of IIa, IIa/IIx and IIx fibers, whereas IIx/IIb and specially type I fibers are minimally affected and (*vi*) no major change in muscle fiber type composition occurs in McA mice during aging.

In this longitudinal study (from 2013 to 2018), we have collected sufficient data (from 139 matings and more than 2,000 offspring mice) to establish that difficulties associated with the management of the McA mouse colony are evident. The difficulties are caused by perinatal and post-weaning mortality of McA mice. Only two cases of fatal infantile form of McArdle disease (16 days and 13 weeks after birth, respectively)^19,20^ associated with hypotonia^19^, respiratory deficiency^19,20^ and generalized weakness^20^ have been reported. In this context, we could not identify the causes for the perinatal death in McA mice and whether these were similar to those involved in the aforementioned fatal infantile forms in patients. However, it is possible that this issue is solvable by splitting litters and adding a lactating female mouse without litters. This may reduce the risk of fighting among littermates for food, which McA mice likely are not well equipped for due to their weaker condition.

We have observed an aging-associated progressive degeneration of muscle fibers, fibrosis and infiltration of inflammatory cells in all the studied muscles. However, this study has demonstrated a differential affection and progression of muscle with age. *Quadriceps* and *soleus* seemed to be less histologically affected by disease progression than *gastrocnemius*, EDL and TA. In the case of the *soleus* this observation was supported by an increase in fiber size and CNF with aging, suggesting that its muscle fibers might be able to withstand repeated cycles of damage and regeneration (this increase was not observed in *gastrocnemius*, EDL, TA and *quadriceps* muscle fibers indicating that these might not be as resistant to progressive damage, leading to their subsequent degeneration); and also by the presence of type I fiber, predominant in the *soleus*, that were almost structurally unaffected in McA mice at all ages analyzed. With respect to *quadriceps*, low fibrosis accumulation and mild fiber size increase during aging suggested that this muscle was less affected by disease progression than *gastrocnemius*, EDL and TA. Furthermore, we had previously reported a seven-fold lower expression of *Pygm* mRNA and GP-M protein in *quadriceps* in comparison to TA^10^. The lower glycogen levels in the *quadriceps* compared to TA muscle of McA mice, points to a moderate use of glycogen metabolism in *quadriceps* in comparison to other glycolytic muscles. The higher content of IIb fibers in *quadriceps* compared to TA and EDL confers an compensatory increase in uptake of glucose for direct glycolysis^11,21–23^; additionally, in rat fiber types it was observed that insulin-directed glucose uptake took place preferentially in IIa fibers (IIa > IIx, IIb), while in IIb fibers glucose uptake was mainly contraction-induced through AMPK mechanisms^24^. Thus, the contraction-induced glucose uptake for direct metabolism rather than glycogen synthesis might spare the *quadriceps* from devastating glycogen accumulation^10^. All these observations clearly indicated that the differential progression of muscle degeneration among glycolytic muscles in McA mice related to their different fiber type composition. In this regard, we previously reported that types I/IIa, IIa, IIa/IIx and IIx muscle fibers presented evident signs of degeneration in McA mice, while type IIx/IIb and specially type I fibers were almost unaffected^10^. In the present study, we have further confirmed these results as type I fibers had an almost normal morphology across all ages in McA mice, whereas there was a progressive degeneration of IIa, IIx and IIa/IIx fibers (*i.e*., from 8 to 70 weeks of age). Additionally, all actively regenerating fibers (*i.e*., MHCe positive) co-stained with IIa, IIx or IIa/IIx fiber type. The reason why type I/IIa, IIa, IIa/IIx and IIx fibers are more affected than type I and IIx/IIb hybrid fibers in the McArdle mouse model is unknown. Yet, a recent study on the proteomics of healthy murine single fiber types^25^ showed a distinct proteomic content among fiber types that might help to explain the observed differences in fiber type resistance to degeneration. In this regard, Mitsugurmin-53/Trim 72, a protein that plays a role in membrane repair, was found to be considerably more abundant in type I than in all another fiber types^25^. Additionally, we previously showed in McA mice that type I fibers presented lower levels of glycogen than type II fibers^6^, and the glycogen content levels in the *soleus* of these mice were lower than in the glycolytic muscles *gastrocnemius* and EDL^7^, suggesting a direct correlation between muscle fiber glycogen content and damage. Selective type II fiber degeneration has been previously reported in other myopathies such as Duchenne muscular dystrophy, facioscapulohumeral muscular dystrophy, myotonic dystrophy type 2^26^ as well as in the alpha-glucosidase knock-out mouse model that mimics the late-onset form of Pompe disease^27,28^. Several reports also indicate that age-related decline in muscle mass is fiber type-specific, principally affecting type II fibers, with type I fibers remaining largely unaffected^25,29–31^. Although no major change in fiber type composition was observed in McA mice during the aging process, we observed a trend towards an increase in glycolytic metabolism in the *soleus* muscle with aging in McA mice, as indicated by a decrease in the percentage of oxidative fibers paralleled by an increase in the proportion of glycolytic fibers in 70-week-old animals. Although the physiologic significance of this observation still needs to be elucidated, it might imply the necessity of this muscle to diversify its metabolism as a protective mechanism against the cytosolic glycogen degradation blockade. Finally, we have shown that progressive muscle degeneration in McA mice during aging is not accompanied by an increase in their muscle glycogen content. This absence of progressive glycogen accumulation was not associated with a re-expression of the *Pygb* and/or *Pygl* genes in mature muscle. Additionally, a significant increase in GS phosphorylation was observed in McA mouse muscles in the three ages analyzed indicating that GS inactivation plays an important role to explain the absence of glycogen content increase. However, some degree of glycogen phosphorylase-independent glycogen degradation cannot be discarded as it remains to be determined whether autophagy-related pathways are progressively involved through aging in McA disease. Although no significant increase in lysosomal GAA was observed in older mice, the enzyme activity might be sufficient to handle potential deviation of glycogen degradation to the lysosomal pathway. Future studies should elucidate whether lysosomal glycogen degradation is affected in McA mice and might determine the link between cytosolic and lysosomal glycogen metabolism in McA disease, as well as its potential therapeutic implications in patients. On the other hand, our finding of an aging-associated progressive degeneration of muscle fibers, fibrosis and infiltration of inflammatory cells in all the studied McA mouse muscles (although with varying degrees of affectation depending on muscle type) is important from a translational point of view. Indeed, we have reported in the Spanish Registry of McA patients [originally n = 239^4^ and n = 333 in the more recent update^5^] that age has a detrimental effect on several phenotypic features of the disease, especially on muscle fixed weakness, which affects mostly proximal/trunk muscles^4^. The mean age (57 ± 19 years) of those patients in the highest severity class 3 (that includes presence of fixed muscle weakness) is indeed higher than in those in the lower severity classes 1 (46 ± 19 years, *p* = 0.007)^5^. Thus, the finding of age-associated muscle fibrosis and infiltration found here might provide mechanistic insight for the reported findings in the older patients and should be kept in mind as an additional hallmark of the disease.

## Supplementary information

Supplementary Info

## Data Availability

The datasets generated during and/or analysed during the current study are available from the corresponding author on reasonable request.
