# Supplementary material for: Low survival rate and muscle fiber-dependent aging effects in the McArdle disease mouse model
Source: Sci Rep. 2019 Mar 26;9:5116. doi: 10.1038/s41598-019-41414-8 (PMC6435661; doi:10.1038/s41598-019-41414-8)
Supplement: Supplementary file 1 — Supplementary Info [file 41598_2019_41414_MOESM1_ESM.pdf]

Low survival rate and muscle fiber-dependent aging effects in the McArdle disease mouse model

Real-Martinez A[1♯], Brull A[2♯], Huerta J[1], Tarrasó G[1], Lucia A[3,4,5], Martin MA[4,5], Arenas J[4,5], Andreu AL[1,5], Nogales-Gadea G[5,6], Vissing J[7], Krag TO[7], de Luna N[8,5♯] and Pinós T[1,5 ♯*]

SUPPLEMENTARY FILES

Supp. Fig. 1

EDL 8 weeks

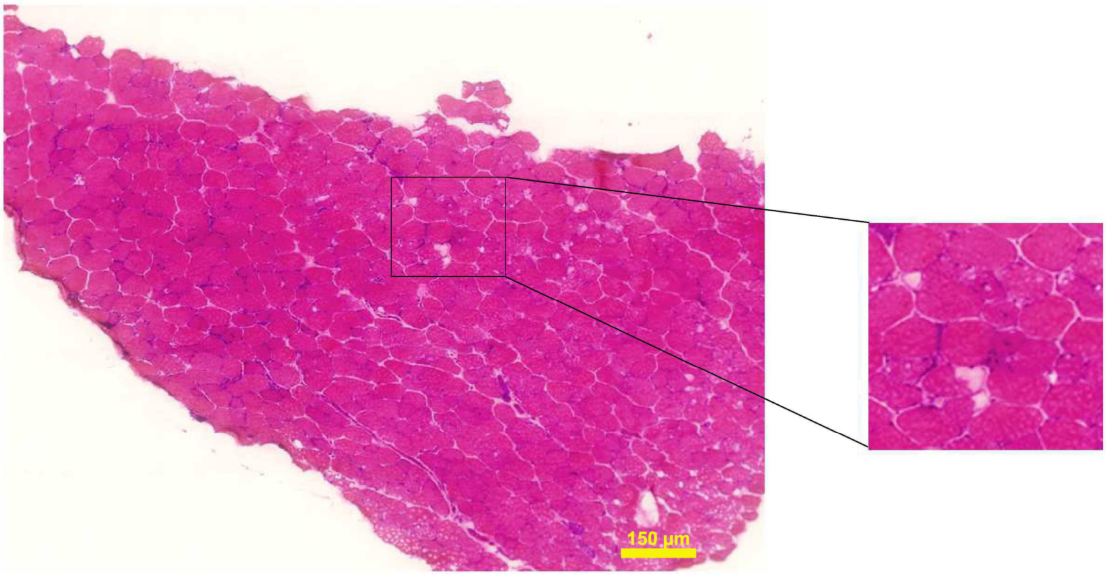

EDL 35 weeks

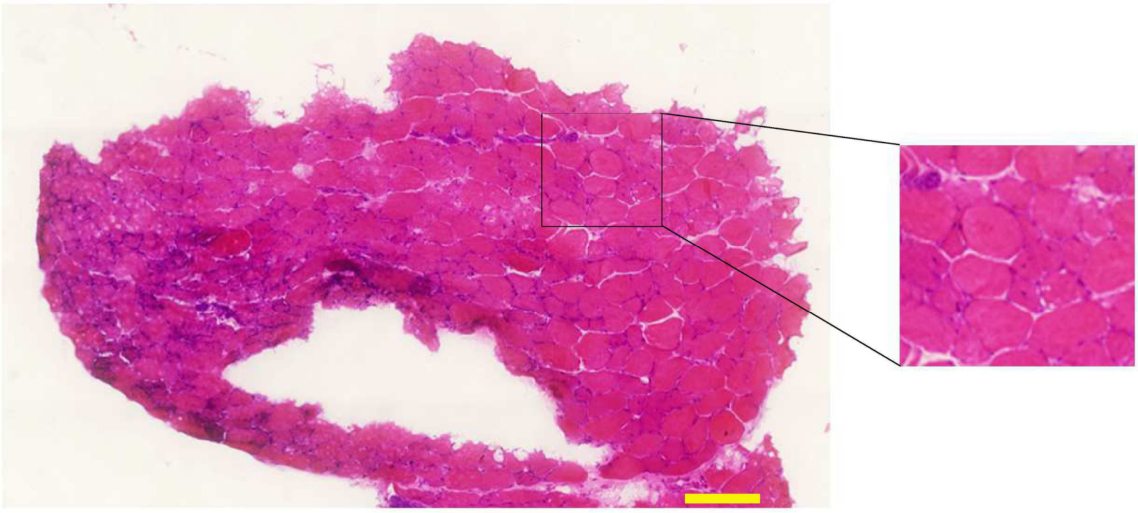

EDL 70 weeks

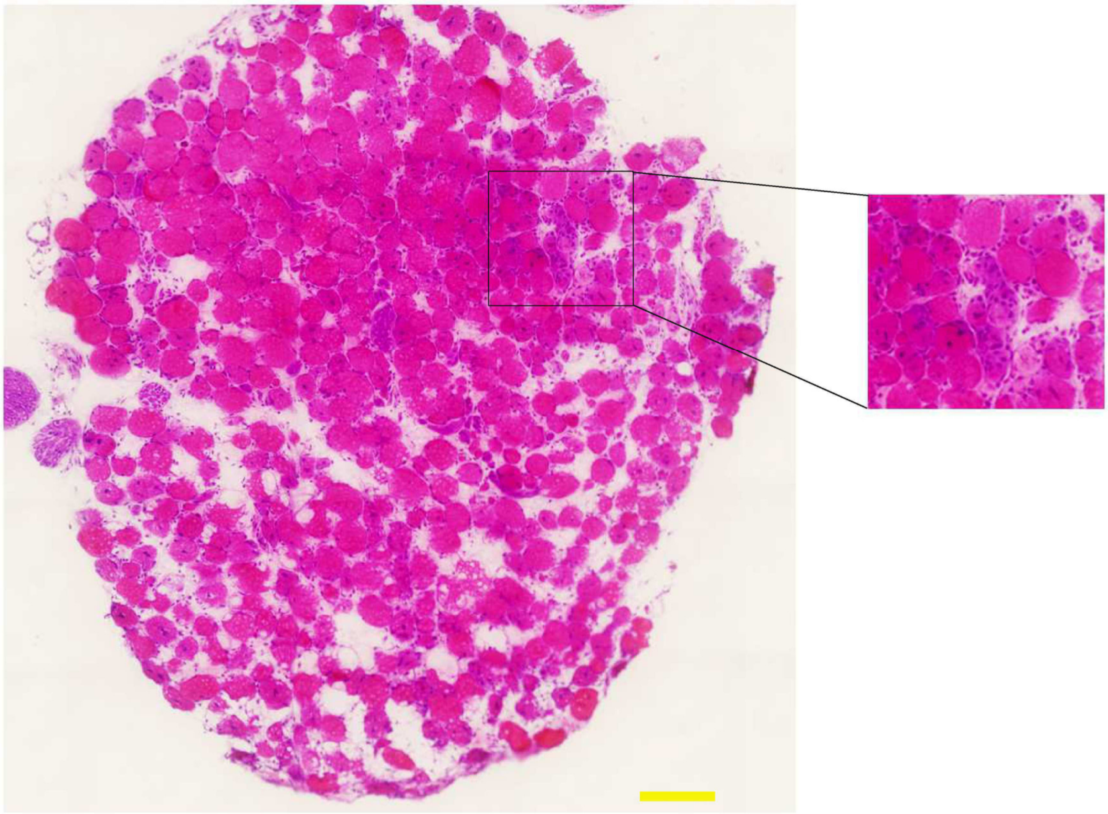

Supp. Fig 2

A

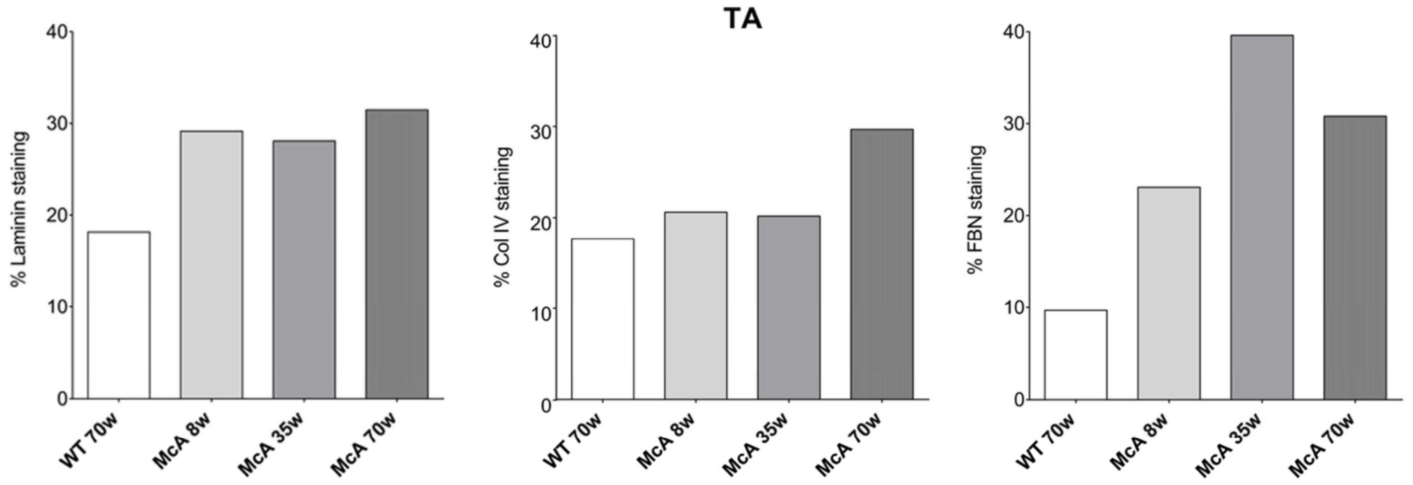
TA
% Laminin staining
% Col IV staining
% FBN staining
0
10
20
30
40
WT 70w
McA 8w
McA 35w
McA 70w

B

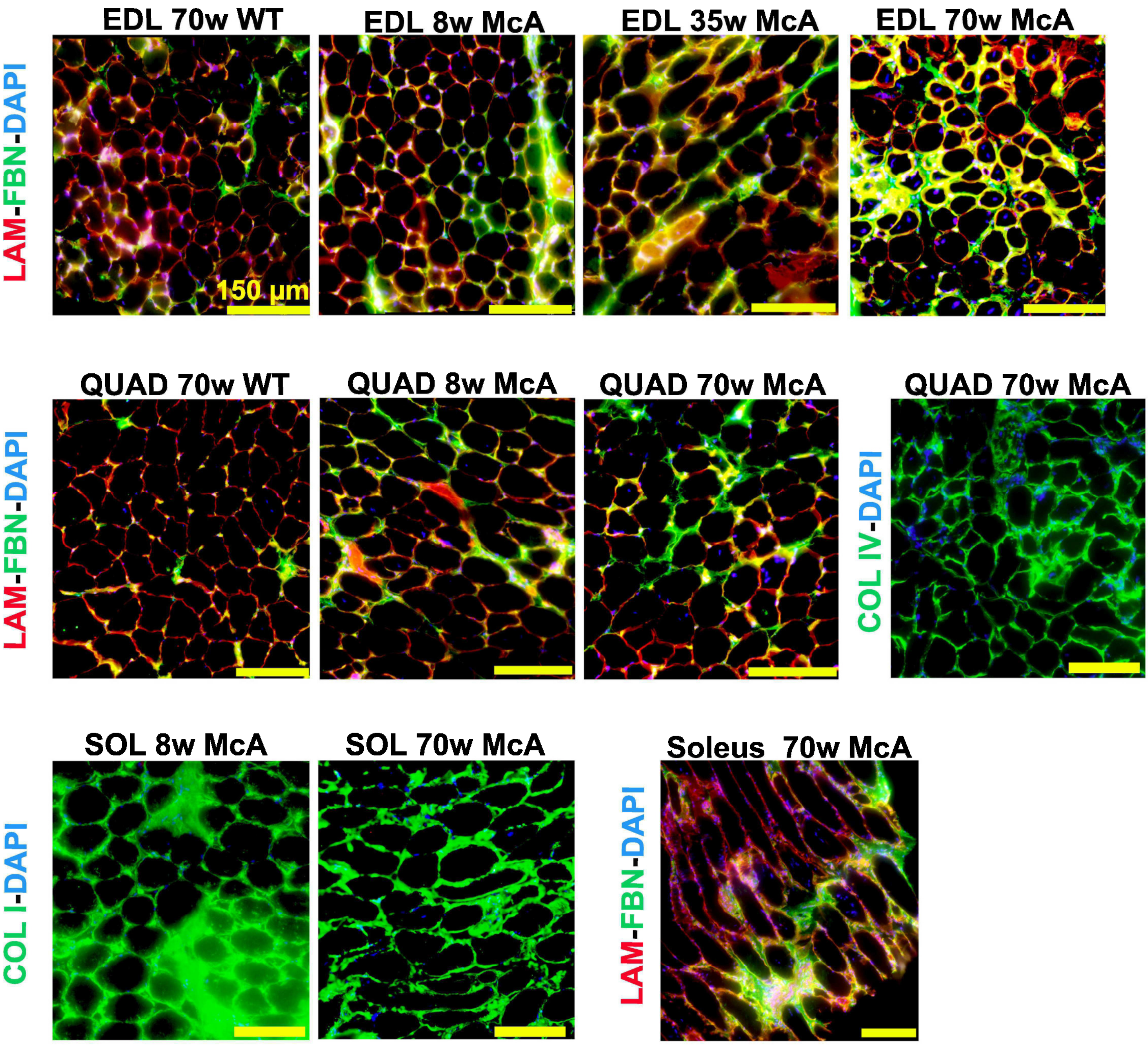
EDL 70w WT
EDL 8w McA
EDL 35w McA
EDL 70w McA
LAM-FBN-DAPI
150 µm
QUAD 70w WT
QUAD 8w McA
QUAD 70w McA
QUAD 70w McA
COL IV-DAPI
SOL 8w McA
SOL 70w McA
Soleus 70w McA
COL I-DAPI

Supp. Fig 3

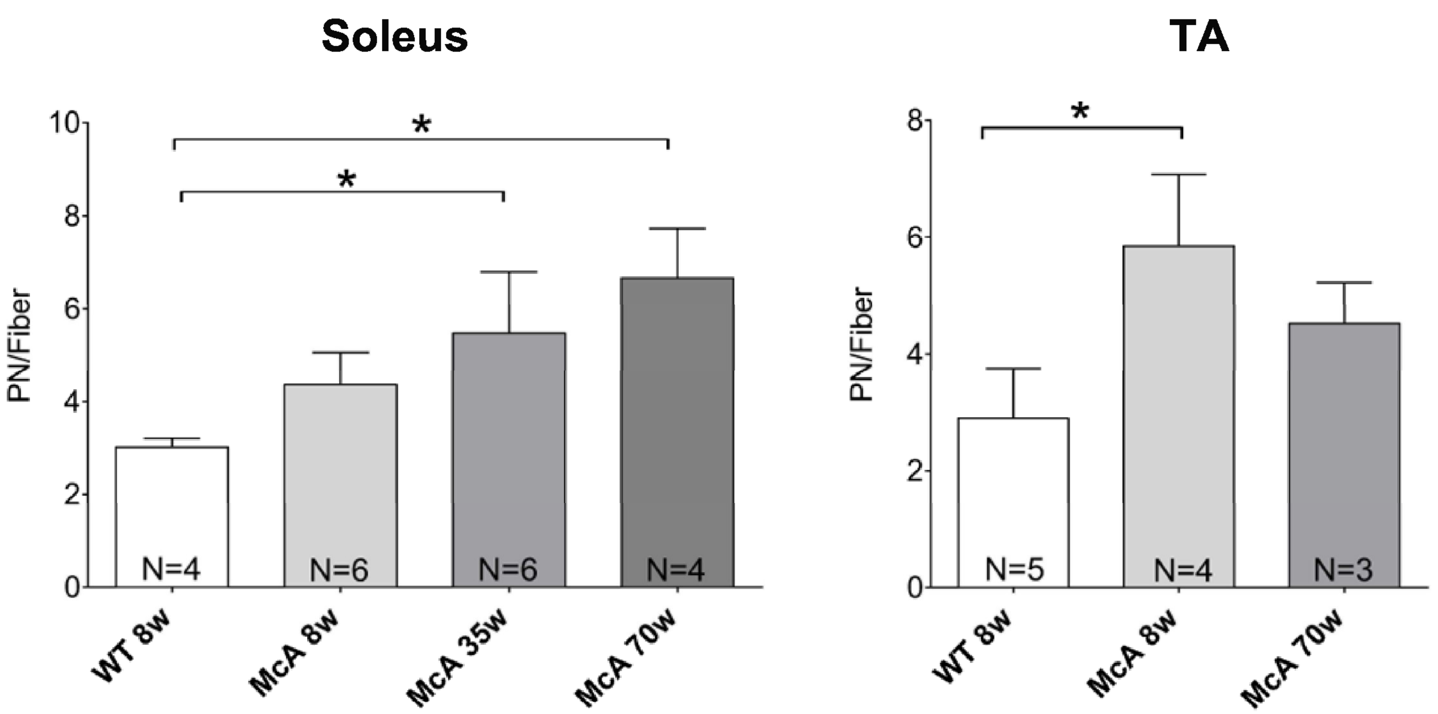
Soleus
PN/Fiber
10
8
6
4
2
0
*
*
N=4
N=6
N=6
N=4
WT 8w
McA 8w
McA 35w
McA 70w
TA
PN/Fiber
8
6
4
2
0
*
N=5
N=4
N=3
WT 8w
McA 8w
McA 70w

Supp. Fig. 4

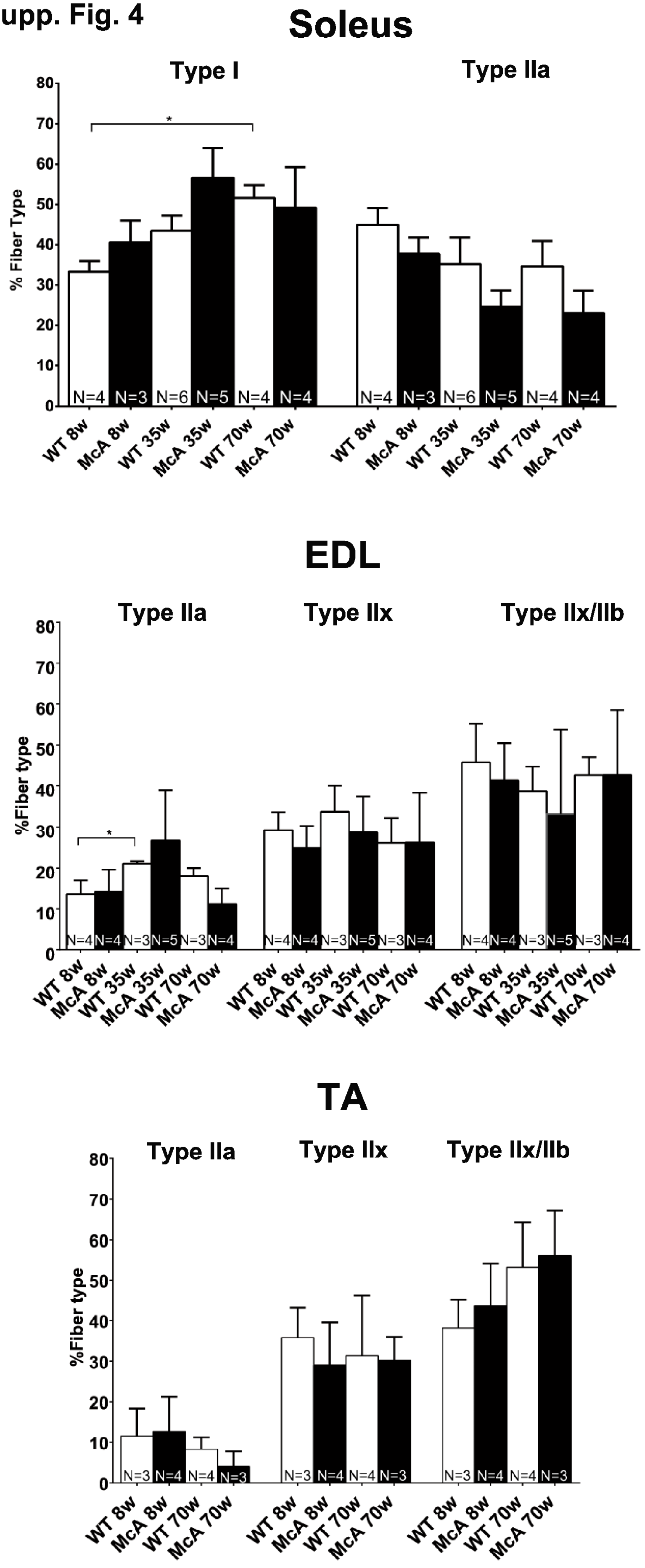
Soleus
Type I
Type IIa
% Fiber Type
0
10
20
30
40
50
60
70
80
*
N=4
N=3
N=6
N=5
N=4
N=4
WT 8w
McA 8w
WT 35w
McA 35w
WT 70w
McA 70w
EDL
Type IIa
Type IIx
Type IIx/IIb
%Fiber type
TA
Type IIa
Type IIx
Type IIx/IIb
%Fiber type

Supp. Fig. 5

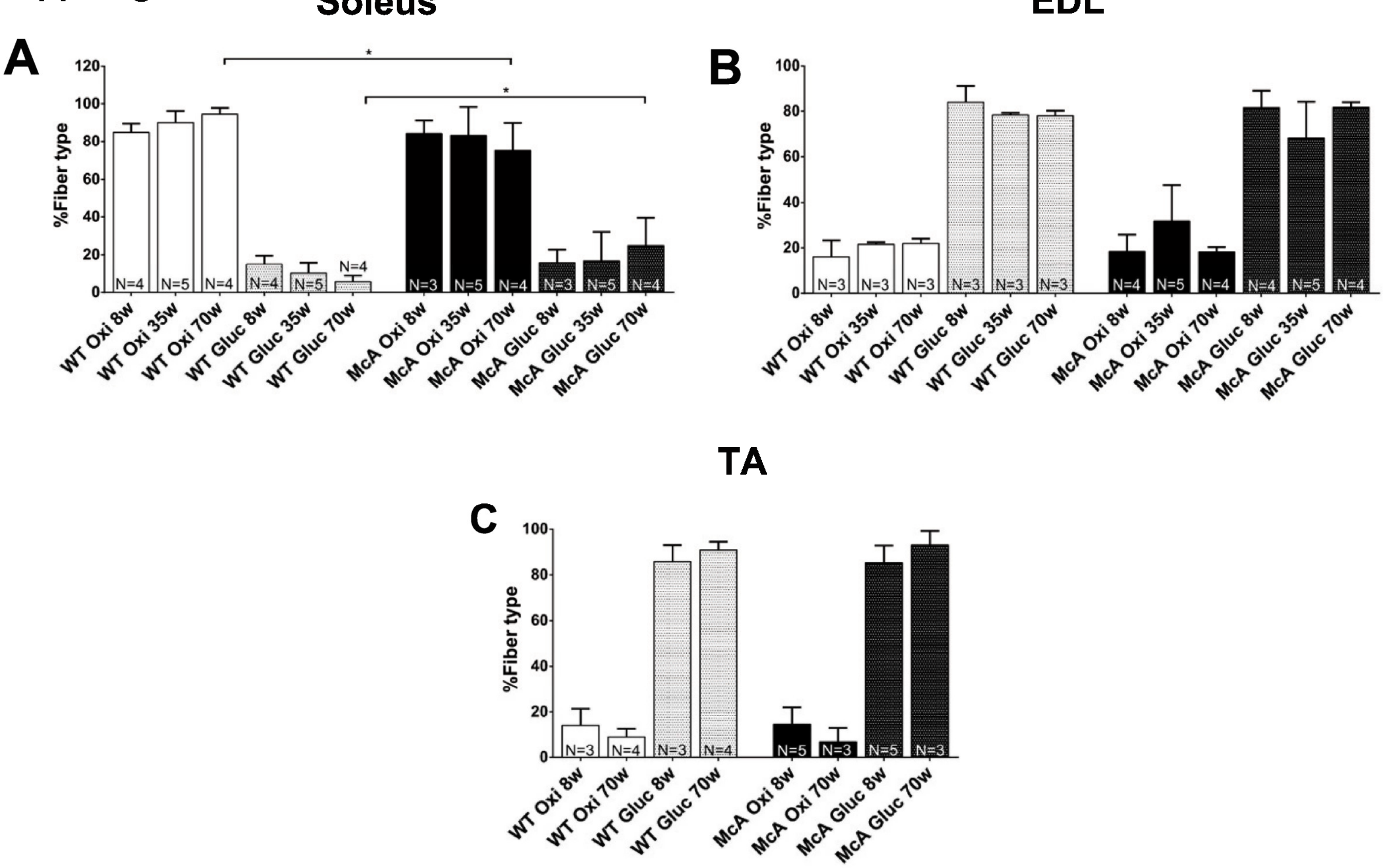
Soleus
A
%Fiber type
120
100
80
60
40
20
0
*
*
N=4
N=5
N=4
N=4
N=5
N=4
N=3
N=5
N=4
N=3
N=5
N=4
WT Oxi 8w
WT Oxi 35w
WT Oxi 70w
WT Gluc 8w
WT Gluc 35w
WT Gluc 70w
McA Oxi 8w
McA Oxi 35w
McA Oxi 70w
McA Gluc 8w
McA Gluc 35w
McA Gluc 70w
EDL
B
%Fiber type
100
80
60
40
20
0
N=3
N=3
N=3
N=3
N=3
N=3
N=4
N=5
N=4
N=4
N=5
N=4
WT Oxi 8w
WT Oxi 35w
WT Oxi 70w
WT Gluc 8w
WT Gluc 35w
WT Gluc 70w
McA Oxi 8w
McA Oxi 35w
McA Oxi 70w
McA Gluc 8w
McA Gluc 35w
McA Gluc 70w
TA
C
%Fiber type
100
80
60
40
20
0
N=3
N=4
N=3
N=4
N=5
N=3
N=5
N=3
WT Oxi 8w
WT Oxi 70w
WT Gluc 8w
WT Gluc 70w
McA Oxi 8w
McA Oxi 70w
McA Gluc 8w
McA Gluc 70w

Supp. Fig 6 **GASTROCNEMIUS** **TA**

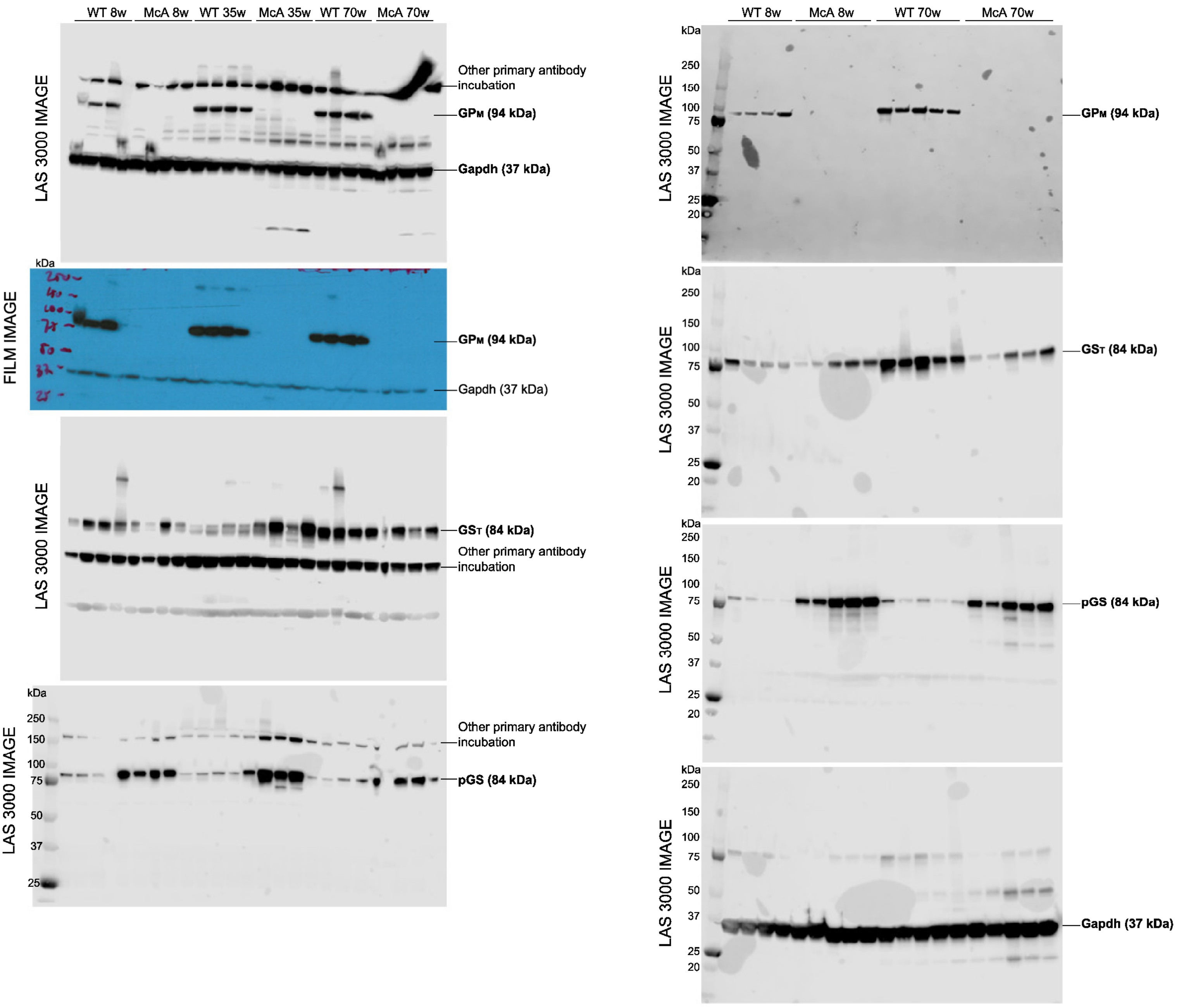

Supp. Table

Primary antibody				Secondary antibody			Application
ID	Reference	Dilution	Diluent	ID	Dilution	Diluent	
Laminin	(MA1-06100/Thermo-Scientific)	1/300	PBS 1x+3% FCS	Goat to Rat Ig H+L (AF 594 nm)	1/500	PBS 1x+3% FCS	IF
Fibronectin	(ab2413/Abcam)	1/200	PBS 1x+3% FCS	Goat to Rabbit Ig H+L (AF 488 nm)	1/500	PBS 1x+3% FCS	IF
Colagen IV	(ab/Abcam)	1/200	PBS 1x+3% FCS	Goat to Rabbit Ig H+L (AF 488 nm)	1/500	PBS 1x+3% FCS	IF
MHC I	(BA-D5/DSHB)	1/20	PBS 1x+3% FCS	Goat to Mouse Ig γ2b (AF 488 nm)	1/500	PBS 1x+3% FCS	IF
MHC IIa	(SC-71/DSHB)	1/20	PBS 1x+3% FCS	Goat to Mouse Ig γ1 (AF 594 nm)	1/500	PBS 1x+3% FCS	IF
MHC IIx	(SAB2104768/Sigma-Aldrich)	1/100	PBS 1x+3% FCS	Goat to Rabbit Ig H+L (AF 488 nm)	1/500	PBS 1x+3% FCS	IF
MHC IIb	(BF-F3/DSHB)	1/30	PBS 1x+3% FCS	Goat to Mouse Ig M (AF 594 nm)	1/500	PBS 1x+3% FCS	IF
MHCe	(F1.652/DSHB)	1/6	PBS 1x+3% FCS	Goat to Mouse Ig γ1 (AF 594 nm)	1/500	PBS 1x+3% FCS	IF
CD 68	(ab955/Abcam)	1/200	PBS 1x+3% FCS	Goat to Mouse Ig γ1 (AF 594 nm)	1/500	PBS 1x+3% FCS	IF
PYGM	From Andrea Martinuzzi	1/2000	TTBS 0,1%+5% Skim milk	Donkey to Goat HRP (SCT sc-2020)	1/5000	TTBS 0,1%+5% Skim milk	WB
GYS-1	(07/2015/Cell Signalling Technology)	1/1000	TTBS 0,1%+5% Skim milk	Goat to Rabbit HRP (Dako P0448)	1/5000	TTBS 0,1%+5% Skim milk	WB
p-GYS-1 Ser 641	(07/2015/Cell Signalling Technology)	1/1000	TTBS 0,1%+5% Skim milk	Goat to Rabbit HRP (Dako P0448)	1/5000	TTBS 0,1%+5% Skim milk	WB
GAPDH	(AM 4300/Ambion)	1/10000	TTBS 0,1%+5% Skim milk	Rabbit to Mouse HRP (Dako P0260)	1/7000	TTBS 0,1%+5% Skim milk	WB
GAA	(ab102815/Abcam)	1/1000	TTBS 0,1%+5% Skim milk	Goat to Rabbit HRP (Dako P0448)	1/5000	TTBS 0,1%+5% Skim milk	WB

Legends:

Supplementary Figure 1. H&E staining of *extensor digitorum longus* (EDL) muscle from 8, 35 and 70-week-old McArdle (McA) mice. All scale bars correspond to 150 µm. A clear increase in interstitial space is observed in 70-week-old McA mice.

Supplementary Figure 2. A) Fibrosis quantification in *tibialis anterior* (TA) muscle using laminin, collagen IV (Col IV) and fibronectin (FBN) staining. Fibrosis was quantified as the percentage of laminin, Col IV and FBN staining in a given muscle area. In each column the percentage of staining was calculated for muscle areas ranging from 1354737 (minimum) to 7409553 (maximum) μm^2. To obtain these areas 2 to 4 different mice were used. B) Laminin-fibronectin double stainings as well as collagen (Col) I and IV single staining in *extensor digitorum longus* (EDL), *quadriceps* and *soleus* of McA mice. All scale bars correspond to 150 µm

Supplementary Figure 3. Number of peripheral nuclei (PN) surrounding the sarcoplasmic membrane per muscle fiber. In each column 490 (minimum) to 1265 (maximum) fibers were counted (median 909 fibers). The non-parametric Kruskal-Wallis *One-Way ANOVA* with multiple comparisons test (*Dunn's test*) was used for statistical analyses. Symbols: * $p<0.05$.

Supplementary Figure 4. A) Percentage of type I and IIa fibers in the *soleus*. In each column between 223 (minimum) and 980 (maximum) fibers were counted (median 612 fibers). B) Percentage of type IIa, IIx and IIx/IIb fibers in the EDL muscle. In each column between 177 (minimum) and 651 (maximum) fibers were counted (median 413 fibers). C) Percentage of type IIa, IIx and IIx/IIb fibers in TA muscle. In each column between 50 (minimum) and 930 (maximum) fibers were counted (median 520 fibers) of each column indicates the number of mice used to calculate the mean values. The N at the bottom of each column represents the number of mice used to calculate the mean

values. Error bars correspond to SD. The non-parametric Kruskal-Wallis *One-Way ANOVA* with multiple comparisons test (*Dunn's test*) was used for statistical analyses. Symbols: * p<0.05.

Supplementary Figure 5. A), B) and B) Percentage of oxidative (type I+IIa+I/IIa) and glycolytic (IIa/IIx+IIa+IIx+IIx/IIb) fibers in 8, 35 and 70-week-old wild-type (WT) and McArdle (McA) mice in *soleus*, EDL and *tibialis anterior* (TA), respectively. The N at the bottom of each column represents the number of mice used to calculate the mean values. Error bars correspond to SD. The non-parametric Kruskal-Wallis *One-Way ANOVA* with multiple comparisons test (*Dunn's test*) was used for statistical analyses. Symbols: * p<0.05.

Supplementary Figure 6. Uncropped original western blot images for GP_M, GS_T, pGS and Gapdh in *gastrocnemius* and *tibialis* anterior (TA) muscles.

Supplementary Table. List of antibodies. Abbreviations. FCS: Fetal Calf Serum; IF: Immunofluorescence; PBS: Phosphate buffered saline; TTBS: Tween-Tris buffered saline; WB: Western blot.
